# T27. DYSFUNCTIONAL ATTITUDES IN ADOLESCENTS WITH EARLY-ONSET PSYCHOSIS: PRELIMINARY RESULTS

**DOI:** 10.1093/schbul/sby016.303

**Published:** 2018-04-01

**Authors:** Olga Puig-Navarro, Francina Badia, Yuli Gallegos, Mireia Forner, Inmaculada Baeza, Gisela Sugranyes, Eva Varela, Anabel Martinez-Aran, Eric Granholm

**Affiliations:** 1 Hospital Clinic of Barcelona; 3 VA San Diego Healthcare System; 4 Hospital Clínic of Barcelona, CIBERSAM; 5 University of California San Diego, VA San Diego Healthcare System

## Abstract

**Background:**

Dysfunctional attitudes such as defeatist performance beliefs (DPB) and asocial beliefs (AB) have been linked to negative symptoms and functional outcome in chronic schizophrenia (Campellone et al., 2016; Grant and Beck, 2010) and in adults with recent onset schizophrenia (Ventura et al., 2014). Adolescents with early-onset psychosis (EOP) are at major risk of having persistent negative symptoms (Puig et al., 2017) but no previous study has examined dysfunctional attitudes in this population. We aimed: (1) to examine if more DPB and AB were present in adolescents with EOP compared with normal controls, and (2) to study the relationships between DPB and AB with symptoms and functioning.

**Methods:**

Sample: 15 adolescents with EOP (11♀, age=15.33 ± 1.23) and 10 healthy controls (8♀, age=15.60 ± 1.51), participants in a trial about cognitive and behavioral social skills treatment in EOP developing in Hospital Clínic of Barcelona (baseline data). Patients were under antipsychotic meds. Inclusion criteria: having an early-onset schizophrenia spectrum disorder diagnosed between 9–18 years-old (schizophrenia, schizoaffective disorder, psychosis n.o.s.); being within the 5 first years after the disease onset; clinical stability. Exclusion criteria: IQ<70; comorbid substance dependence disorder; neurological disorders. Instruments: PANSS (Kay et al., 1987), Calgary Depression Scale (CDS, Addington et al., 1990), Children’s Global Assessment Scale (C-GAS, Shaffer et al., 1983), Social and Role Functioning Scales (GF:S, GF:R, Cornblatt et al., 2007), Life Skills Profile - Adolescent version (Puig et al., 2013), Dysfunctional Attitudes Scale - Spanish version (DAS, Sanz et al., 1993); Asocial Beliefs Scale (Grant and Beck, 2010). DPB was extracted from the DAS scale following Beck et al. (2013). Groups were compared with T-test or Chi-square tests. Pearson correlations were computed for examining relationships between variables.

**Results:**

EOP and control groups were homogeneous in age (t=-0.49, p=0.632) and sex (X2=0.15, p=0.702). All subjects were living with their parents. Family SES was lower in EOP group (X2=10.69, p=0.030). All subjects but one patient were studying although patients had repeated more courses (t=4.00, p=0.001). EOP subjects had a predominance of negative symptoms (positive symptoms=14.07 ± 3.85; negative symptoms=23.50 ± 13.79; general symptoms=31.86 ± 7.28). Mean score of CDS in EOP was low (3.77 ± 4.76). EOP group had lower scores in all functional measures than controls (C-GAS: 51.14 ± 9.38 vs 93.70 ± 4.45, GF:S: 5.71 ± 1.07 vs 9.00 ± 0.47, GF:R: 5.21 ± 1.12 vs 8.80 ± 0.42, LSP: 67.64 ± 12.30 vs 44.13 ± 3.60; t>6.67, p<0.001). Regarding dysfunctional attitudes, EOP group had higher scores in dysfunctional attitudes scales than healthy controls (DPB: 55.60 ± 19.57 vs 33.00 ± 9.37, AB: 6.67 ± 3.42 vs 3.00 ± 2.11; t>3.02, p<0.006). In EOP, DPB and AB scores were negatively correlated with functioning (DPB: C-GAS r=-0.60, GF:S r=-0.66, GF:R r=-0.66, LSP r=0.65; p<0.002; AB: C-GAS r=-0.45, GF:S r=-0.55, GF:R r=-0.49, LSP r=0.53; p<0.029). No significant correlation were found between dysfunctional attitudes and negative symptoms in EOP.

**Discussion:**

These preliminary results showed that adolescents with EOP had higher levels of dysfunctional attitudes than controls. Accordantly to recent literature, defeatists performance beliefs and asocial beliefs were correlated with poorer functioning. However, dysfunctional attitudes were not associated with negative symptoms. It might be that dysfunctional attitudes do not contribute to negative symptoms but in functional outcome in youngs with EOP although larger samples are required. Acknowledgments: Fundación Alicia Kolplowitz.

